# Assessing Psychosocial Distress in Cystic Fibrosis: Validation of the ‘Distress in Cystic Fibrosis Scale’

**DOI:** 10.1007/s10880-021-09825-w

**Published:** 2021-09-29

**Authors:** Caroline Finlay, Sejal Patel, Jonathan Evans

**Affiliations:** 1 Acute Psychology, NHS Greater Glasgow and Clyde, Glasgow, UK; 2 Institute of Health and Wellbeing, University of Glasgow, Glasgow, UK; 3 Pain Management Psychology, Victoria Infirmary, 55 Grange Road, Glasgow, G42 9LF UK

**Keywords:** Adult, Cystic fibrosis, Psychological distress, Measurement

## Abstract

Experiences of anxiety and depression are common in adults with Cystic Fibrosis (AwCF) (e.g. Quittner in Thorax 69:1090-1097, 2014) and may impact on a wide range of important health-related behaviours, such as adherence to medication and timely attendance for medical review when experiencing pulmonary exacerbation. Common screening measures used in CF such as the PHQ-9 and GAD-7 may reflect an absence of anxiety or depression when clinically significant emotional difficulties are apparent on further assessment. This study preliminarily validated the previously developed Distress in Cystic Fibrosis Scale (DCFS) (Patel in Journal of Cystic Fibrosis 15:S26, 2016); a 23-item questionnaire to assess psychosocial distress in AwCF. Inpatient and outpatient participants with CF (*N* = 119) completed a battery of questionnaires, including the DCFS. PCA results supported a single component model. The DCFS showed high internal consistency and correlated significantly with measures of mood and quality of life. The DCFS shows promise as a screening tool to assess clinically significant psychosocial distress in an adult CF population.

## Introduction

Cystic Fibrosis (CF) is an inherited, progressive and life-limiting condition in which the lungs and digestive system become obstructed by thick, sticky mucus. Many organ systems are affected in the body, but it is the frequent pulmonary exacerbations—resulting in progressive decline in lung functioning—that has the greatest impact on mortality. CF is caused by mutations in the cystic fibrosis transmembrane conductance regulator (CFTR) gene. Recent medical advances involving the newest CFTR modulator therapies (Heijerman et al., 2019) bring significant hope for reduced number of chest infections and improved survival for those people with CF who are genetically eligible. The ‘triple combination therapy’ (brand name ‘Kaftrio’ in Europe; ‘Trikafta’ in the US) has been shown to reduce pulmonary exacerbations, improve lung function, increase Body Mass Index (BMI) and improve subjective respiratory symptoms for people with CF who have at least one of the most common CF gene mutations (Delta F508) (Middleton et al., 2019). This accounts for more than 70% of people worldwide with CF. Despite these advances, for many patients, CF still results in a significant physical and emotional burden, particularly in light of COVID-19 and the level of threat this posed to particularly clinically vulnerable groups. Adults with chronic conditions are at higher risk of experiencing depression and/or anxiety compared to community samples (Yang et al., 2013; Smith & Schmitz, 2014). These rates may be two to three times higher in adults with CF (AwCF) than those without (Quittner et al., 2014). Depressive symptoms in AwCF are associated with poor health-related quality of life and poor health outcomes (Riekert et al., 2007); increased healthcare utilisation and costs (Snell et al., 2014); and poor treatment adherence (Knudsen et al., 2016). The International Committee on Mental Health in Cystic Fibrosis (ICMH-CF) (Quittner et al., 2016) recommends the use of the 9-item Patient Health Questionnaire (PHQ-9) (Kronke & Spitzer., 2002) and 7-item Generalised Anxiety Disorder Questionnaire (GAD-7) (Spitzer et al., 2006) to screen once a year for depression and anxiety, respectively. Whilst useful in this context, detecting a wider range of psychosocial difficulties in AwCF is clinically pertinent (Oxley & Webb, 2005). For example, ‘normal’ scores on the PHQ and GAD measures may be achieved by AwCF for whom managing treatments, attending clinic, or having an annual review causes significant distress and affects management of the condition.

CF ‘annual reviews’ are a yearly appointment offered to patients for a consultation with each discipline of the specialist CF multidisciplinary team (MDT). In the UK, the CF MDT usually includes at least one CF specialist in each of the following disciplines; pharmacy, dietetics, physiotherapy and clinical psychology, in addition to clinical nurse specialists and respiratory physicians. In the US, social work is a required part of the annual review process. Patients receive various medical tests and scans during an annual review, including blood tests and a full pulmonary function test which may require the patient to sit inside a fairly small glass box. The entire annual review process can take a number of hours, may show results that the patient finds distressing (e.g. a drop in lung function) and can feel invasive and uncomfortable. Patients may avoid such appointments due to emotional distress, but they are a vital part of monitoring and surveillance to maintain good CF health.

Pakhale et al. (2015) found that in addition to general mood and anxiety difficulties, AwCF may wish to discuss treatment adherence, quality of life concerns, death and difficulties with stigma/disclosure to others. In a physical health context the term ‘psychosocial distress’ has been used to highlight the broad array of difficulties that individuals may experience (Holland, 1997). There is a growing movement towards a paradigm shift beyond medicalisation and diagnosis in mental health and towards a multi-factorial and contextual approach (The Power Threat Meaning Framework (PTM) (Johnstone and Boyle, 2018). Understanding the distress that is related to different aspects of CF is an important development in keeping with this evidence-based paradigm shift.

Disease-specific measures of psychosocial distress are available for other long-term conditions (Hoffman et al., 2004; Polonsky et al., 2005). To our knowledge, no validated measures of psychosocial distress in an adult CF population currently exist. The Distress in Cystic Fibrosis Scale (DCFS) (Patel, 2016) was previously developed to assist in assessing clinically relevant emotional distress in AwCF. Over a three-year period, 150 patient files were audited to ascertain the emotional concerns AwCF presented with to the CF clinical psychologist. Thirty themes of psychosocial concerns were identified of which eight were excluded due to being isolated occurrences (e.g. domestic violence and perceptual disturbance). The remaining 22 themes were included in the developed questionnaire; constructed by adapting the framework of previously validated measures of distress in long-term conditions, for example, the Distress Thermometer (Hoffman et al., 2004) and the Diabetes Distress Scale (Polonsky et al., 2005). It was presented to CF outpatients during face-to-face contact, and to the CF MDT to check face validity, with positive feedback received. Finally, the questionnaire was shown to the UK Psychosocial Professions in CF (UKPP-CF) group; a network of over 200 psychosocial professionals, where suggestions were made for format improvements and the addition of one further item.

In keeping with psychosocial distress measures in other physical health conditions, the aim of the DCFS is to identify areas of difficulty pertinent to people with CF, where further assessment and intervention may be beneficial, to enhance clinical practice. This study aimed to complete an initial evaluation of the structure and psychometric properties of the DCFS to inform recommendations for future use.

## Methods

Participants (*N* = 119) were recruited from the West of Scotland Adult Cystic Fibrosis Service (WoSACFS) between December 2018 and March 2019, by a familiar clinician either at a routine CF clinic appointment or during in-patient admission. Eligible participants were aged 18 or over and fluent in English. Individuals who had a learning disability or who were inpatient and too acutely unwell to participate were excluded from the study. Interested participants completed paper forms of the research documents or took them away to post back using a pre-paid envelope.

### Measures

BMI, lung function measurements and current bacterial growth were recorded from participants’ medical files where consent was provided. In CF, bacterial flora in the lungs is routinely recorded for treatment and segregation purposes. Since microbial status may have significant bearing on various aspects of CF-related quality of life, it was selected as an important measure. Due to the potential for cross-infection, CF clinics and inpatient accommodation at WoSACFS are largely segregated according to three categories of pathogen growth; ‘non-cepacia’, ‘cepacia’ and M.abscessus, which were the categories recorded in this study.

The DCFS (Patel, 2016) is a 23-item, self-report questionnaire with a response ranging between 0 (‘no problems’) and 10 (‘worst I’ve ever felt’) (see appendix 1). Respondents were instructed to indicate how they had been feeling over the past two weeks relating to each of the 23 items. Some items were not relevant to everyone therefore ‘N/A’ responses were coded as such in SPSS so as to differentiate from genuine missing data.

The PHQ-8 (Kroenke et al., 2009) and the GAD-7 (Spitzer et al., 2006) are widely used and validated self-report measures of depression and anxiety, respectively. Responses are based on how the individual has been feeling over the past two weeks and use a four-point scale from ‘not at all’ to ‘nearly every day’. The PHQ-8 was selected instead of the PHQ-9 to omit the final question regarding suicidality for the purposes of safety and monitoring during the research study.

The Cystic Fibrosis Questionnaire—Revised (CFQ-R; Quittner et al., 2000) is a 50-item health-related quality of life (HRQOL) measure for children, adolescents and adults with CF. Results using the adult form provide standardised scores on nine quality of life domains (physical functioning, role limitations, energy/well-being, emotional state, social limitations, body image, eating disturbances, treatment constraints, overall health perception) and three symptom scales (weight, respiratory symptoms and digestive symptoms). The CFQ-R has robust psychometric properties (Quittner et al., 2012).

Finally, participants were asked to rate how much each questionnaire covered their current difficulties using a four-point scale from ‘did not cover any of my difficulties’ to ‘covered all of my difficulties’. Participants also rated how easy or difficult each questionnaire was to complete using a five-point scale from ‘very difficult’ to ‘very easy’.

### Data Analysis

Principal Component Analysis (PCA) was conducted to explore the structure of the DCFS. Internal consistency was tested using Cronbach’s Alpha. Criterion validity was investigated by exploring the extent to which the DCFS scores correlated with the other validated measures and also the ability of the DCFS to discriminate between those experiencing psychosocial distress and those who were not. Exploratory and descriptive statistics were conducted in relation to content validity, particularly relating to the practical usage of the DCFS and the wording of the instructions and response scale. Finally, descriptive statistics were used to evaluate participants’ ratings of the questionnaires. Missing data were coded as such in SPSS and all analyses were run with pairwise deletion where possible so as to maximise sample size and power.

## Results

### Participant Demographics

Table 1 summarises demographic and clinical characteristics of participants. As expected, the majority of participants were characterised with ‘non cepacia’ pathogen growth and participated as outpatients.

**Table 1:** Demographic and clinical characteristics

Demographic and clinical characteristics (* N* = 119)	Mean (SD)
Age	30.7 (11.1)
BMI	22.6 (3.4)
FEV ^1^%	67.1 (30.1)
Clinical measures	
Personal health questionnaire depression scale (PHQ 8)	5.4 (5.1)
Generalised anxiety disorder questionnaire (GAD-7)	4.6 (4.5)
Distress in cystic fibrosis scale (DCFS)	19.6 (16.3)
Cystic fibrosis questionnaire-revised (CFQ-R)—(*range across twelve domains*)	51.4–84.7 (18.3–37.3)
	*n* (%)
Gender	
Male	68 (57.1)
Female	51 (42.9)
Pathogen growth category	
Non-cepacia (includes all strains of Pseudomonas)	99 (83.2)
*B. cepacia* (*Burkholderia cepacia*)	12 (10.1)
*M. abscessus* (*Mycobacterium abscessus*)	8 (6.7)
Setting	
Outpatient	92 (77.3)
Inpatient	27 (22.7)

### Principal Component Analysis

PCA was run on the 23-item DCFS. Inspection of the correlation matrix showed that all variables had at least one correlation coefficient greater than 0.3. The overall Kaiser Meye-Olkin (KMO) measure was 0.83, with individual KMO measures all greater than 0.6. Bartlett’s test of sphericity was statistically significant (*p* < 0001), indicating that the data were likely factorisable. PCA revealed five components that had eigenvalues greater than one and which explained 37.3%, 7.5%, 7.4%, 6.1%, and 5.3% of the total variance, respectively. The five-component solution explained 63.7% of the total variance but after applying Direct Oblimin rotation the rotated solution did not exhibit a simple or meaningful structure. Subsequent exploratory PCAs were conducted, with the two and four component solutions exhibiting the simplest structure, but meaningful interpretation continued to be difficult. On further inspection of the extraction criteria, the first component had an Eigen value of 8.6, with the remaining four components having Eigen values between 1 and 1.7. Additionally, the scree plot clearly demonstrated one-component before the inflection point. Consequently, a one-component solution was extracted, with all items loading strongly, providing support to retain all items.

### Internal Consistency

The 23-item DCFS was found to have high internal consistency (*α* = 0.913 *n* = 65), with a range for the total scale, as measured by alpha if item-deleted, between 0.905 and 0.914. However, in this analysis ‘N/A’ responses were considered to be missing data resulting in the analysis using only 50% of the study population. To overcome this, ‘N/A’ responses were re-coded as ‘0’ (given that N/A does mean that there was no distress relating to that item) and the analysis rerun. High internal consistency (*α* = 0.911 *n* = 119) was again demonstrated, with a range for the total scale, as measured by alpha if item-deleted, between 0.902 and 0.912.

### Criterion Validity

Based on theoretical and empirical considerations, a series of associations between DCFS items and previously validated measures were chosen a priori. Table 2 illustrates that all correlations were in the predicted direction, and all met statistical significance criteria (*p* < 0.05), supporting DCFS criterion validity.

**Table 2:** Summary of a priori chosen correlations

	DCFS Q1^a^	DCFS Q2^b^	DCFS Q7^c^	DCFS Q9^d^
FEV^1^	Rho = − 0.26, *p* = .007 *N* = 107			
PHQ-8 total		Rho = 0.73, * p* < .001 *N* = 118		
GAD-7 total		Rho = 0.74, * p* < .001 *N* = 119		
CFQ-R physical	Rho = − 0.66, * p* < .001 *N* = 118			
CFQ-R emotion		Rho = − 0.76, * p* < .001 *N* = 118		
CFQ-R social			Rho = − 0.47, * p* < .001, * N* = 118	
CFQ-R eating				Rho = − 0.57, * p* < .001, * N* = 118

*CFQ-R* cystic fibrosis questionnaire-revised, *DCFS* distress in cystic fibrosis scale, *FEV*^1^ forced expiratory volume in 1 s, *GAD-7* generalised anxiety disorder questionnaire, *PHQ-8* personal health questionnaire depression scale

^a^_DCFS Q1 = How have you been feeling physically?_

^b^_DCFS Q2 = How have you been feeling emotionally?_

^c^_DCFS Q7 = How have you been feeling about your relationships with other people?_

^d^_DCFS Q9 = How have you been feeling about your body, weight and/or eating?_

There were strong correlations between DCFS Q2 and existing measures of mood and quality of life. The data were then split into those who scored seven or above on at least one item on the DCFS and those who did not. A cut-off score of seven was selected to ensure that the group represented those who rated themselves as experiencing levels of distress at the higher end of the scale. Differences between the groups on PHQ-8 and GAD-7 total scores were investigated. As expected, participants who had scored seven or above in at least one item on DCFS had higher PHQ-8 and GAD-7 scores than those who scored below seven on all items. However, the mean PHQ-8 and GAD-7 scores for the group who had scored seven or more on one item were 8.8 and 7.4, respectively, which did not meet clinical cut-off point of 10. Overall these analyses suggest that the DCFS is able to pick up difficulties detected by the PHQ-8 and GAD-7, but it is also able to detect additional distress that is not identified by the PHQ-8 or GAD-7.

The ability of the DCFS to discriminate between those scoring above and below clinical cut-off point (10) on PHQ-8 and GAD-7 was evaluated. A Mann Whitney test revealed DCFS total scores for ‘depressed’ group (mean rank = 95.81) were significantly higher than for ‘non-depressed’ group (mean rank = 49.49), *U* = 275, *z* = − 6.12, *p* < .001, *ɳ*^*2*^ = 0.32. A further Mann Whitney test revealed DCFS total scores for ‘anxious’ group (mean rank = 95.75) were significantly higher than for ‘non-anxious’ group (mean rank = 51.89), *U* = 280.5, *z* = − 5.39, *p* < .001, *ɳ*^*2*^ = 0.25. This suggests that the DCFS is able to discriminate between those scoring above and below the clinical cut-off for GAD-7 and PHQ-8.

### Content Validity

**Fig. 1:**
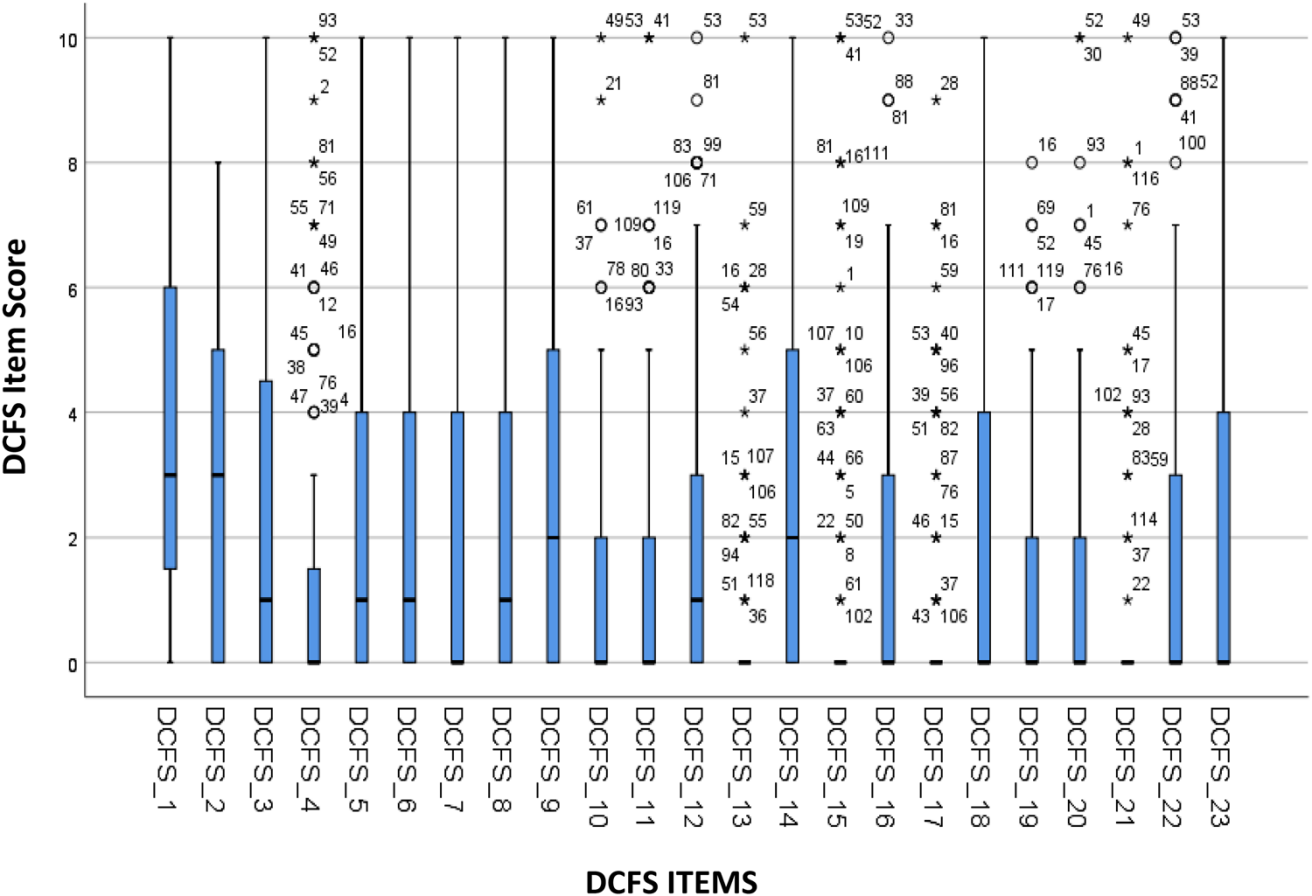
Boxplots for all DCFS items

The method used to create the DCFS (see Patel, 2016) supports content validity of the screening tool. Visual inspection of boxplots (Fig. 1) revealed responses were skewed towards the lower end of the distress scale, with medians for all items being under three. For four items only outliers were presented as up to 80% of participants responded ‘0’ or ‘N/A’. However, for every item, including those with only outliers, the boxplots illustrate a range of distress ratings provided by participants from ‘0’ to at least ‘8’, with the majority of items having scores of ‘10’ by several participants. These descriptive analyses provide support for the ‘0–10’ scale and for all items to be included in the DCFS.

### DCFS Items

Further exploratory analyses were conducted to investigate the distribution of responses across all items and to consider the wording of the response scale. For items relating to specific physical health symptoms there was a high percentage (60–70%) of participants rating these as ‘0’. Due to the wording of ‘no problems’ being associated with the score of ‘0’ on the visual scale at the top of the questionnaire it is unclear whether participants were reporting ‘no distress’ in that area as intended, or whether they meant that the specific item did not apply to them. In Q22 regarding ‘upsetting past events’ only one participant recorded this as ‘N/A’, whilst 73 (61.3%) recorded it as ‘0’. It may be unlikely that these individuals have all experienced significant previous upsetting events but do not have any current distress in that area. These data suggest that participants are possibly interchanging between ‘N/A’ and ‘0’ responses, particularly on certain physical health items.

### Questionnaire Evaluation

74% of participants rated the DCFS as covering most or all of their difficulties compared to 64% for the PHQ-8 and 60% for GAD-7. With regards to ease of completion, all questionnaires were rated as easy or very easy by the majority of participants.

## Discussion

Despite recent medical advances in the treatment of CF (Heijerman et al., 2019), clinical experience suggests that emerging physical health improvements may not correlate with significant psychosocial improvement. Furthermore, the triple combination is not available to patients in every country and—outwith the National Health Service (NHS)—there are associated costs making the treatment prohibitively expensive. Within those who are eligible and who can access the medications, other difficulties may prevent long-term use such as medical complications. This study was conducted prior to the approval of the triple combination therapy in Scotland, the advent of which has impacted in complex ways on the emotional well-being of AwCF taking the drugs. Anecdotally, distress related to adjustment reactions to various bodily and life changes following treatment with triple therapy may be an emerging problem. The COVID-19 pandemic will have had further impact on the distress of AwCF who have been required to take stringent measures in order to protect themselves from the threat of infection.. Screening for psychosocial distress in a CF population—beyond the constructs of depression and anxiety—remains a significant priority.

The established model of CF psychological service delivery requires regular ‘surveillance’ for every patient in addition to the usual contacts with inpatients and outpatients for assessment and therapeutic intervention. Frequent and brief consultations during inpatient admissions and MDT clinics (which occur every three months in the UK) allow for a working formulation and essential therapeutic relationship building; for example to assess and intervene in problems with adherence, or patients declining required treatment. A brief tool to detect the common emotional difficulties that may affect AwCF would be beneficial in this model of care.

The PHQ-9 and GAD-7 are recommended screening measures in CF (Quittner et al., 2016). They can alert all CF clinicians to significant emotional difficulties at annual review, and can be used as useful outcome measures for clinical psychology interventions. However, many clinical psychologists may work within less ‘diagnostic’ frameworks, for example in line with the ideas presented in the PTM Framework (Johnstone and Boyle, 2018). Furthermore, people with CF may present with clinically significant difficulties that are not detected by traditional anxiety and depression measures. One example is attendance at MDT clinic; an essential requirement for all people with CF. It involves the person with CF being offered a consultation with each speciality of the CF MDT, every 3 months. The appointment may include tests and scans, and can take up to 2 h. People with CF with an absence of anxiety or depression as measured by the GAD or PHQ tools, may nevertheless experience significant distress about this particular event, resulting in avoidance of attending. Screening for distress about attending CF MDT clinic can alert the CF clinical psychologist to an area of important psychological support, with improving attendance, coping with lung function results, or uptake of suggested treatments as examples of therapeutic goals.

The present study validated a brief measure of psychosocial distress in an adult CF population, initially developed to assist CF clinical psychologists conducting assessments or brief reviews. It is expected that the tool can assist in screening for important psychosocial difficulties that people with CF may face, and which affect their ability to manage their condition, but which are not necessarily detected by standard measures of ‘depression’ and ‘anxiety’. A one-component structure for the DCFS emerged, providing support for its use as a measure of psychosocial distress. All items were rated by participants using the full breadth of the ‘0–10’ scale, which gave support to retaining all items as a clinically useful tool. Positive findings relating to internal consistency, criterion validity and participants’ feedback were found. Potential changes to improve content validity are proposed, specifically regarding the instructions and wording of the response scale.

The DCFS was able to accurately identify individuals for caseness using PHQ and GAD measures, with further exploratory analyses highlighting a group of participants for whom the DCFS picks up additional psychosocial distress not detected by current measures. This reflects the concerns raised by clinicians in CF services and strengthens the rationale for a psychosocial screening tool specific to people with CF. With regards to content validity, it is possible that the wording of ‘0’ as ‘no problems’ on the visual ruler at the top of the questionnaire was confusing for participants, resulting in ‘0’ responses being used to indicate that particular issue was not currently relevant to the individual. This also raised the possibility that individuals were only rating distress if the item directly related to a current issue. However, in CF services, it is beneficial to consider the distress related to potential future problems, considering the clinical likelihood of inpatient admissions and deterioration in physical health over time.

Changes are proposed for the future use of the DCFS to add a sentence to the instructions indicating that all the items may or may not directly affect the AwCF now, or in the future, but that we are interested in how they *feel* about the item. This concept is reflected in proposed changes to certain items also, in addition to minor changes to improve readability. A further change is proposed to the wording of the visual scale from ‘no problems’ to ‘no concerns’ and for the ‘N/A’ option to be removed in order to further reinforce that the questionnaire is exploring distress about—and not just the presence of—the item in question. See appendix 2.

### Limitations

This study validated the DCFS using the responses of adults with CF living in the west of Scotland. In order to achieve a more robust validation of the measure, greater diversity in sampling is preferable. The study was also conducted prior to two highly significant global events affecting people with CF; namely COVID-19 and the advent of ‘triple therapy’. Further validation in the wake of these events would be prudent. The items in which only outlier data points were presented on the boxplots could potentially be removed as these items might not be common issues associated with significant distress. However, these items were related to specific physical health symptoms and with the current sample being skewed towards a healthy outpatient population, participants with these difficulties may have been under-represented. The study sample included a higher number of outpatients and those in the ‘non cepacia’ category; participants who may be from a healthier CF population, skewing the distress levels in the study towards the lower end. A wider sample of AwCF, with more equal distributions of adults with different microbial status and lung function—including more inpatients—would be recommended in future studies.

## Conclusion

The current study suggests the DCFS is a promising screening tool for detecting psychosocial distress in an adult CF population. It is able to detect issues that existing validated measures identify, whilst also identifying additional difficulties that were undetected by previous measures. Furthermore, it provides helpful detail about the areas of psychosocial distress of concern. Participants’ feedback suggests it is an accessible tool and they value an opportunity to think about the emotional impact of CF. Having a user-friendly, quick tool to detect these difficulties as early as possible would allow timely further assessment and appropriate intervention to be provided. These initial findings of the utility of the DCFS are promising and recommendations have been made regarding possible changes. Further validation studies are recommended. This study was conducted prior to the COVID-19 pandemic, and prior to the introduction of the triple combination CFTR modulator therapies. These have had significant impact on the CF population and the DCFS might usefully be developed further to incorporate these as further items.

## Data Availability

Original data set can be provided upon request.

## Distress in cystic fibrosis scale

Below is a list of common issues affecting people with Cystic Fibrosis.

- Please read each item carefully, and using the scale at the top as a guide, fill in the box next to each question with the number that comes closest to how you have been feeling about that issue ***over the past 2 weeks***.
- If a question doesn’t apply to you (e.g. for Q. 10, if you don’t have Diabetes) just write in N/A for ‘not applicable’.
- Please don’t think over each question too much; just write the number that first comes to mind.

**Table:**

0	1	2	3	4	5	6	7	8	9	10
No problems										Worst I’ve ever felt

	Over the past 2 weeks	0...10
1.	How have you been feeling** physically?** (E.g. feeling tired, in pain, chesty, blocked up or anything else)	
2.	How have you been feeling** emotionally? ** (E.g. feeling sad, worried, angry, upset or anything else)	
3.	How have you been feeling** about your work situation? ** (Whether or not you do paid work)	
4.	How have you been feeling** about your housing situation? **	
5.	How have you been feeling** about your financial situation? ** (E.g. debts/ benefits)	
6.	How have you been feeling **about going out, socialising, or having things to do in the day?**	
7.	How have you been feeling** about your relationships with other people?** (E.g. your partner, friends, family or anyone else)	
8.	How have you been feeling** about managing CF treatments?**	
9.	How have you been feeling** about your body, weight and/or eating?**	
10.	How have you been feeling** about your Diabetes control?**	
11.	How have you been feeling** about having a specific procedure or treatment?** (E.g. getting a button, a port, blood tests/ needles, or anything else)	
12.	How have you been feeling** about lung function tests and results? **	
13.	How have you been feeling** about coughing up blood (haemoptysis)?**	
14.	How have you been feeling** about CF getting worse?**	
15.	How have you been feeling** about a recent (new) diagnosis or bug?**	
16.	How have you been feeling** about fertility, pregnancy or parenting?**	
17.	How have you been feeling** about anything to do with transplant? ** (E.g. just thinking about it, being on the list, or having had one already)	
18.	How have you been feeling** about being in hospital?**	
19.	How have you been feeling** about coming to clinic?**	
20.	How have you been feeling** about telling other people about CF?** (E.g. at work, school, college or university, or friends & family)	
21.	How have you been feeling** about your use of street drugs or alcohol?**	
22.	How have you been feeling** about upsetting past events? ** (E.g. memories of an accident, experiences as a child, a medical procedure, or anything else)	
23.	How have you been feeling** about anything to do with end of life?** (E.g. Someone you know who has died, or concerns about when you die)	

## Distress in cystic fibrosis scale (DCFS)

Below is a list of common issues that affect many people with CF. They may or may not all directly affect you now, or in the future. We are still interested in how you feel about them.

- Please read each item carefully, and using the scale at the top as a guide, fill in the box next to each question with the number that comes closest to how you have been feeling about that issue ***over the past 2 weeks***.
- Please don’t think over each question for more than a few seconds; just write in the first number that comes to mind.

**Table:**

0	1	2	3	4	5	6	7	8	9	10
No concerns										Worst I’ve ever felt

	Over the past 2 weeks	0...10
1.	How have you been feeling** physically?** (E.g. feeling tired, in pain, chesty, blocked up or anything else)	
2.	How have you been feeling** emotionally? ** (E.g. feeling sad, worried, angry, upset or anything else)	
3.	How have you been feeling** about work ? ** (Whether or not you do paid work)	
4.	How have you been feeling** about where you live? **	
5.	How have you been feeling** about money? ** (E.g. debts/ benefits)	
6.	How have you been feeling **about going out, socialising, or having things to do in the day?**	
7.	How have you been feeling** about your relationships with other people?** (E.g. your partner, friends, family or anyone else)	
8.	How have you been feeling** about managing CF treatments?**	
9.	How have you been feeling** about your body, weight and/or eating?**	
10.	How have you been feeling **about CF-related diabetes (CFRD)?** (Either now, or as a potential future possibility)	
11.	How have you been feeling** about having a specific procedure or treatment?** (E.g. getting a button, a port, blood tests/ needles, or anything else)	
12.	How have you been feeling** about lung function tests and results? **	
13.	How have you been feeling **about coughing up blood (haemoptysis)?** (Either now, or as a potential future possibility)	
14.	How have you been feeling** about CF getting worse?**	
15.	How have you been feeling** about a recent (new) diagnosis or bug?**	
16.	How have you been feeling** about fertility, pregnancy or parenting?**	
17.	How have you been feeling** about anything to do with transplant? ** (E.g. just thinking about it, being on the list, or having had one already)	
18.	How have you been feeling **about being in hospital?** (Either now, or as a potential future possibility)	
19.	How have you been feeling** about coming to clinic?**	
20.	How have you been feeling** about telling other people about CF?** (E.g. at work, school, college or university, or friends & family)	
21.	How have you been feeling** about using street drugs or drinking alcohol?**	
22.	How have you been feeling** about upsetting past events? ** (E.g. memories of an accident, experiences as a child, a medical procedure, or anything else)	
23.	How have you been feeling** about anything to do with end of life?** (Your own, someone you care about, or just in general)	
